# Childhood adiposity trajectories are associated with late adolescent blood pressure: birth to twenty cohort

**DOI:** 10.1186/s12889-016-3337-x

**Published:** 2016-07-29

**Authors:** Richard J. Munthali, Juliana Kagura, Zané Lombard, Shane A. Norris

**Affiliations:** 1 School of Molecular and Cell Biology, Faculty of Science, University of the Witwatersrand, The Mount, 9 Jubilee Road, Parktown, Johannesburg, 2193 South Africa; 2 Sydney Brenner Institute for Molecular Biosciences (SBIMB), University of the Witwatersrand, Johannesburg, South Africa; 3 MRC/Wits Developmental Pathways for Health Research Unit (DPHRU), University of the Witwatersrand, Johannesburg, South Africa; 4 Division of Human Genetics, School of Pathology, Faculty of Health Sciences, University of the Witwatersrand and National Health Laboratory Service, Johannesburg, South Africa

**Keywords:** Blood pressure, Latent classes, Latent class growth mixture modeling, Body mass index trajectories, Trajectories, Hypertension, Childhood adiposity, Obesity

## Abstract

**Background:**

Elevated blood pressure in childhood is a risk factor for adult hypertension which is a global health problem. Excess adiposity in childhood creates a predisposition to develop adult hypertension. Our aim was to explore distinct sex-specific adiposity trajectories from childhood to late adolescence and examined their association with blood pressure.

**Methods:**

Latent Class Growth Mixture Modeling (LCGMM) on longitudinal data was used to derive sex-specific and distinct body mass index (BMI: kg/m^2^) trajectories. We studied 1824 black children (boys = 877, girls = 947) from the Birth to Twenty (Bt20) cohort from Soweto, South Africa, and obtained BMI measures at ages 5 through 18 years. Participants with at least two age-point BMI measures, were included in the analysis. Analysis of variance (ANOVA), chi-square test, multivariate linear and standard logistic regressions were used to test study characteristics and different associations.

**Results:**

We identified three (3) and four (4) distinct BMI trajectories in boys and girls, respectively. The overall prevalence of elevated blood pressure (BP) was 34.9 % (39.4 % in boys and 30.38 % in girls). Boys and girls in the early onset obesity or overweight BMI trajectories were more likely to have higher BP values in late adolescence. Compared to those in the normal weight BMI trajectory, girls in early onset obesity trajectories had an increased risk of elevated BP with odds ratio (OR) of 2.18 (95 % confidence interval 1.31 to 4.20) and 1.95 (1.01 to 3.77). We also observed the weak association for boys in early onset overweight trajectory, (*p*-value = 0.18 and odds ratio of 2.39 (0.67 to 8.57))

**Conclusions:**

Distinct weight trajectories are observed in black South African children from as early as 5 years. Early onset adiposity trajectories are associated with elevated BP in both boys and girls. It is important to consider individual patterns of early-life BMI development, so that intervention strategies can be targeted to at-risk individuals.

## Background

Hypertension is a global public health problem affecting more than one billion people globally [1]. It is a risk factor for developing cardiovascular disease (CVD) and also contributes to an increase in mortality worldwide. A recent systematic analysis reported that hypertension accounts for about nine million deaths globally every year [2]. In African countries, 25 % of deaths are caused by hypertension [1]. Hypertension is also one of the leading causes of heart attacks, stroke and kidney failure [2]. Results from the Heart of Soweto Study reported a 55 % hypertension prevalence among adult South Africans aged 52.8 years on average [3]. Some studies have looked at elevated blood pressure (BP) in childhood, mostly in rural South Africa, with reported prevalence rates ranging from 1 to 25.9 % [4–7].

Previous studies have reported that childhood blood pressure weight gain from childhood to adulthood, adulthood obesity [8, 9], childhood and adolescent physical in-activity and certain lifestyle behaviors such as use of tobacco and alcohol consumption [10], are some of the determinant factors of adulthood hypertension. Understanding the life-course progression of adiposity in children is important since childhood adiposity is associated with adult obesity which has been reported to be linked to increased hypertension risk in adults [11]. Kagura and colleagues reported that elevated BP can be observed from childhood in black urban South African children [12]. Most of the studies that reported on childhood obesity and adult hypertension have used cross sectional data. There are few studies that have used longitudinal data to understand the effect of life-course childhood adiposity on late adolescent blood pressure and lack of comprehensive longitudinal data have made it difficult to do such studies in an African setting.

There has been increased interest in the role of growth patterns on BP, it is also still unclear how early childhood adiposity influences later BP [13]. Using longitudinal data will help elucidate this relationship and this study in particular will add further evidence from an African perspective. For the first time in an exclusively African population, we sought to determine whether adiposity progression (from 5 to 18 years of age), focused on using body mass index (BMI) as an adiposity marker, could be used to predict BP among late adolescents.

We use a relatively new method in both biology and epidemiology called Latent Class Growth Mixture Modeling (LCGMM) to identify distinct sex-specific adiposity trajectories. LCGMM groups individuals with the same developmental trajectory in the same class. It also allows for inclusion of variation in several growth parameters both within and between classes [14]. Developmental trajectories of BMI describe individuals’ BMI change over time. One of the advantages for using LCGMM is that adiposity trajectories are identified independently without pre-assumptions of the relative contributions of various lifestyles, genetic and epigenetic factors. Therefore multiple factors may contribute to variation in the information of these adiposity trajectories.

The aims of this study were to: 1) determine the distinct sex-specific patterns of adiposity trajectories in black South Africa children from 5 to 18 years of age, 2) find the prevalence of elevated blood pressure in late adolescence, and 3) to explore associations between these distinct adiposity trajectories to elevated BP in late adolescence.

## Methods

### Participants

To identify developmental patterns of BMI from 5 to 18 years old and relate them to blood pressure in late adolescence, data from the Birth to Twenty cohort (Bt20) were used. Bt20 is Africa’s largest and longest running longitudinal birth cohort, with 3273 children at time of enrolment. It is focused on the health and development of children born in a South African urban township, namely Soweto in Johannesburg. The cohort comprised of 1682 girls and 1591 boys of which the majority were black children (78.4 %). The participants have been followed up to date using different means of communication. The detailed cohort profile with recruitment and cohort attrition details has been described elsewhere [15]. Only black participants (*n* = 1824) with weight and height data available for at least two time points between 5 and 18 years were included in this study.

### Measures

#### Anthropometrics

Trained research assistants collected anthropometric measurements. Weight was measured using a digital scale to the nearest 0.1 kg with participants in light clothes and without shoes. A wall-mounted stadiometer (Holtain, UK) was used to measure standing height to the nearest 0.1 cm. Weight and height at 5 years and 7–18 years old were used to calculate BMI (weight (kg)/height (m^2^)), used as a marker for adiposity at corresponding years.

### Blood pressure assessment

Blood pressure (mm Hg) was measured using Omron 6 automated machine (Kyoto, Japan) from 8 years of age onwards and a Dinamap Vital Signs monitor 1846SX (Critikon, USA) was used at 5 years of age. At each assessment, participants’ seated blood pressure was measured three times with a 2 min interval between each measurement. The blood pressure measurements were taken after 5 min of seated rest. The mean average for the second and third right arm readings was recorded for the current analysis. Blood pressure measurements from one visit have been used in blood pressure studies before [12, 13, 16, 17].

The mean arterial pressure (MAP) was calculated from systolic blood pressure (SBP) and diastolic blood pressure (DBP) using the formula; MAP = (SBP + (2 * DBP))/3. We followed a standard procedure as recommended by the fourth National High Blood Pressure Education Program working group on high blood pressure in children and adolescents (NHBPEP) [18] report to classify blood pressure either normotensive (BP less than 90th percentile), prehypertension or hypertension (BP equal or over 90th percentile for sex, age and height) for those participants who were above 17 years but not yet 18 years old the time BP data was collected. For participants who were already 18 years or older we used the cut-offs as recommended in the seventh report of the Joint National Committee on Prevention, Detection, Evaluation, and Treatment of High Blood Pressure [19]. Prehypertension was defined as SBP readings from 120 to 139 mm Hg or a DBP from 80 to 89 mm Hg while hypertensive was defined as SBP readings equal or greater than 140 mm Hg or DBP readings equal or greater than 90 mm Hg. Due to small sample size the prevalence of hypertension, prehypertension and hypertension were combined and defined as elevated blood pressure.

### Statistical analysis

Different group based modeling methods such as Latent Class Growth Analysis (LCGA) and Latent Class Growth Mixture Modeling (LCGMM), have been used to identify and classify individuals into different trajectories. LCGA technique was developed by Nagin and colleagues [20–23] and is implemented through SAS Proc Traj in the SAS software. There is also a Traj Stata software plugin developed by Jones and Nagin, 2012 [24]. On the other hand LCGMM was developed by Muthén and colleagues and is implemented in Mplus [25–28]. We used Mplus and LCGMM since it allows variation in the intercept and slope in both within the class (inter-individual differences) and across classes while SAS Proc Traj allows only across classes variation. Using LCGMM adds more heterogeneity in the model, thus being the more flexible method as such was preferred in the current analysis [14, 22, 25, 26, 28–30].

The exploratory analyses to check the patterns of missing data concluded that data was missing at random. Mplus handles missing data by the expectation-maximization algorithm (EM algorithm) with assumption of missing at random (MAR) [28]. This makes use of full information maximum likelihood estimation with missing values (FIML) by using estimation to integrate all available information based on MAR assumptions. This means that we can make full use of all available data in our analysis. This prevents the inclusion of only those participants who have no missing data at all data points, which would subsequently reduce the sample size. So the use of the maximum likelihood (ML) approach implemented in Mplus overcomes potential biases in participants with substantial missing data [25, 26, 28, 31, 32].

We included only those participants with at least two time points of available data for BMI in the current analysis. Those with one time point of available data were excluded as this may have influenced the identification and pattern of the BMI trajectories. On average, seven available data points per participant were used to perform this longitudinal analysis.

Latent Class Growth mixture modeling (LCGMM in Mplus version 7.3 [28] was performed to identify the distinct sex-specific BMI trajectories between 5 and 18 years.

The BMI values were used in this study because they are more sensitive to changes in body composition and have been recommended over BMI z-scores [33–35].

Once BMI trajectory group membership was determined for both girls and boys, each respondent was assigned to the class according to the highest probability of belonging to that class. The remainder of analyses was conducted using Stata version 13 [36] to examine additional descriptive characteristics of the classes. We used both standard logistic and multivariate linear regressions to estimate the relationship between latent class membership and blood pressure in late adolescence. Analysis of variance (ANOVA) and chi-square test results were used to assess the differences in different study characteristics among the BMI trajectory classes at 5 % level of significance.

## Results

### BMI Trajectories: optimal number of BMI trajectories

Using LCGMM, we explored linear, quadratic and cubic slopes to fit the model. The cubic slope coefficients were either not significant or required prohibitive running times and did not converge in all models. This suggests that the cubic time function model did not fit the data well. After applying the criteria as recommended by other authors [14, 25, 26, 29, 30, 37–39], only quadratic models involving quadratic time function were further analyzed.

To determine the optimal number of latent classes, we used different model fit indices in conjunction with other criteria as used in previous studies [14, 30, 37, 40, 41]. Firstly, we looked at the three model statistics, the Bayesian Information Criterion (BIC) [42], the Bootstrap Likelihood Ratio Test (BLRT) [43] and Lo, Mendell and Rubin Likelihood Ratio Test (LMR-LRT) [44]. Some studies have applied BIC in variable modeling analyses [41, 42, 45, 46]. It estimates a model to be true using posterior probabilities. A lower BIC would indicate that a model is more likely to be considered as the true model [42]. BIC reduces the false positive rate, hence most individuals will be assigned in their right BMI latent class [47]. We also looked at the entropy value and number of study participants per class. We employed a less restrictive 1 % group membership in this study, which has also been used before in other studies [41, 48]. Lastly we looked at the shape of the BMI trajectories and its clinical interpretability.

Model fit statistics used to come up with optimal number of classes in both boys and girls are shown in Table 1. Applying the criterion discussed above a four-class model fits our data well in girls and a three-class solution has the best classification of BMI trajectory membership in boys.

**Table 1:** Model fit statistics for quadratic LCGMM comparing solutions for 1 to 7 latent classes

Sex	Latent classes	BIC^a^	LMR-LRT^b^ (*P*-value)	BLRT^c^ (*P*-value)	Convergence	Entropy value	Log Likelihood	Number of parameters
Boys	1	23605.4	N/A	N/A	Yes	N/A	−11738.3	19
	2	22846.6	0.03	0.00	Yes	0.98	−11345.4	23
	**3** ^**d**^	**22644.4**	**0.13**	**0.67**	**Yes**	**0.99**	**−11230.7**	**27**
	4	22544.7	0.53	1.00	Yes	0.98	−11167.3	31
	5	22476.5	0.77	0.67	Yes	0.93	−11119.7	35
	6	22438.4	0.61	1.00	Yes	0.94	−11087.1	39
	7				NO			43
Girls	1	27949.8	N/A	N/A	Yes	N/A	−13909.8	19
	2	27226.2	0.00	0.00	Yes	0.94	−13534.3	23
	3	27155.0	0.10	0.00	Yes	0.76	−13485.0	27
	**4** ^**d**^	**27083.9**	**0.23**	**0.00**	**Yes**	**0.80**	**−13435.7**	**31**
	5	27026.0	0.45	0.6	Yes	0.82	−13393.1	35
	6	26990.9	0.63	1.00	Yes	0.78	−13361.8	39
	7	26961.2	0.27	1.00	Yes	0.80	−13333.3	43

*LCGMM* latent class growth mixture modeling

^a^Bayesian Information Criterion

^b^Lo, Mendell and Rubin Likelihood Ratio Test

^c^Bootstrapped Likelihood Ratio Test, N/A – not applicable

^d^The optimal class solutions according to the model fit criteria are shown in bold

**Fig. 1:**
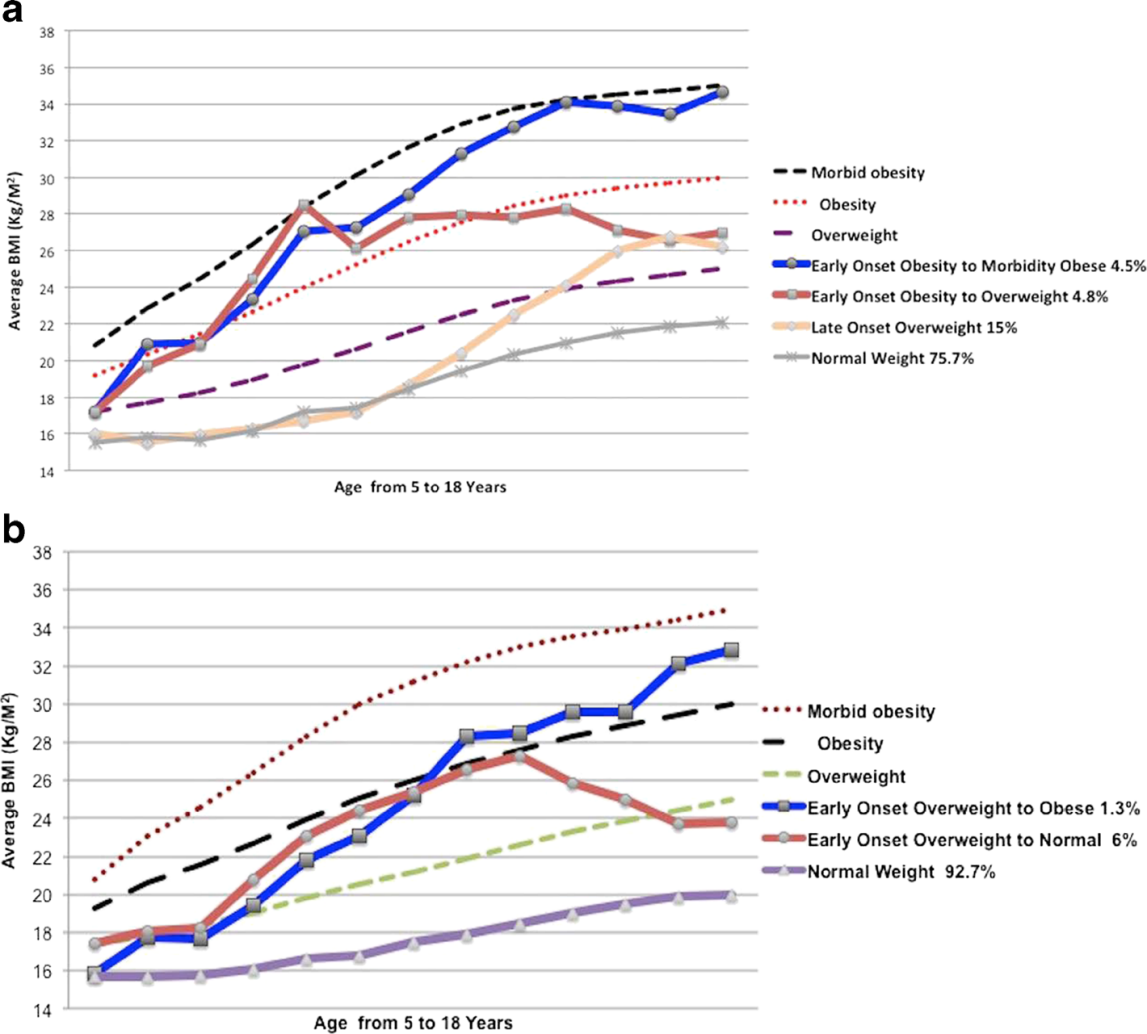
**a** BMI Trajectories in girls. BMI latent classes plotted together with Extended International Obesity Task Force (IOTF) Cut-Offs for BMI in girls. **b** BMI Trajectories in boys. BMI latent classes plotted together with Extended International Obesity Task Force (IOTF) Cut-Offs for BMI in boys

In order to facilitate interpretation of the results, Fig. 1a and b show the BMI trajectory classes for 947 girls and 877 boys in relation to the extended International Obesity Task Force (IOFT) [49]. For girls, four BMI trajectory classes can be described as: Trajectory 1 (normal weight: 75.7 %), Trajectory 2 (late onset overweight: 15 %), Trajectory 3 (early onset obesity to overweight: 4.8 %) and lastly Trajectory 4 (early onset obesity to morbidly obese: 4.5 %). For boys, a three-class model fits the data well. These classes can be classified as: Trajectory 1 (normal weight: 92.7 %), Trajectory 2 (early onset overweight to normal: 6 %) and Trajectory 3 (the early onset overweight to obese trajectory: 1.3 %).

Study characteristics by BMI trajectory classes are shown in Tables 2 and 3. Most of the study characteristics were significantly different among BMI trajectories in girls. In boys, lower maternal education and SBP at 18 years were significantly different among the BMI trajectories and the rest were not.

**Table 2:** Study characteristics by BMI trajectory in girls: The Birth to Twenty Cohort

	Normal weight (*n* = 717)	Trajectory 2 (*n* = 142)	Trajectory 3 (*n* = 45)	Trajectory 4 (*n* = 43)	*P* value
Study characteristics					
Birth weight (kg)	3.0 (0.5)	3.0 (0.5)	3.0 (0.5)	3.3 (0.5)	0.01*
Height at 18 years (cm)	159.7 (6.2)	158.4 (5.1)	157.3 (6.6)	159.3 (6.1)	0.03*
SBP at 18 years (mm Hg)	114.3 (9.6)	116.0 (9.5)	117.9 (11.7)	120.1 (11.3)	0.00***
DBP at 18 years (mm Hg)	71.5 (8.3)	72.8 (8.1)	75.8 (12.7)	75.8 (9.0)	0.00***
MAP at 18 years (mm Hg)	85.8 (7.9)	87.2 (7.8)	89.8 (11.8)	90.6 (8.9)	0.00***
Elevated BP n (%)	187 (28.5)	28 (31.1)	17 (43.6)	18 (46.1)	0.03*

For girls: Trajectory 2 = Late Onset Overweight, Trajectory 3 = Early Onset Obese to Overweight, Trajectory 4 = Early Onset Obese to Morbidity Obese

Summary statistics presented as mean (±standard deviation) otherwise stated

*P < 0.05; **P < 0.01; ***P < 0.001

ANOVA and chi square test were conducted to describe the study characteristics differences in BMI trajectories

Percentages may not add up to 100 % due to rounding

**Table 3:** Study characteristics by BMI trajectory in boys: the birth to twenty cohort

	Normal weight (*n* = 813)	Trajectory 2 (*n* = 53)	Trajectory 3 (*n* = 11)	*P* value
Study characteristics				
Birth weight (kg)	3.1 (0.5)	3.2 (0.6)	3.2 (0 .7)	0.46
Height at 18 years (cm)	170.6 (6.7)	172.4 (5.5)	170.4 (7.6)	0.21
SBP at 18 years (mm Hg)	121.0 (10.8)	124.2 (8.2)	134.5 (16.9)	0.00***
DBP at 18 years (mm Hg)	71.0 (8.6)	71.9 (8.3)	76.8 (8.7)	0.09
MAP at 18 years (mm Hg)	87.7 (8.1)	89.4 (7.3)	96.0 (10.3)	0.00**
Elevated BP n (%)	276 (38.7)	20 (46.5)	6 (60.0)	0.24

For boys: Trajectory 2 = Early Onset Overweight to Normal Weight, Trajectory 3 = Early Onset Overweight to Obese

Summary statistics presented as mean (±standard deviation) otherwise stated

*P < 0.05; **P < 0.01; ***P < 0.001

ANOVA and chi square test were conducted to describe the study characteristics differences in BMI trajectories

Percentages may not add up to 100 % due to rounding

### Blood pressure in late adolescence and BMI trajectory classes

Only participants with blood pressure values at 18 years of age were included in this analysis. It included 766 boys and 823 girls. The overall prevalence of elevated blood pressure in late adolescence was 34.91 % (39.43 % boys and 30.38 % girls). Birth weight and height at 18 years were added in the model to determine whether BMI trajectory classes have an effect on SBP, DBP, MAP and elevated BP status as shown in Tables 4 and 5. In girls, Trajectory 3 was significantly associated with SBP, DBP and MAP with mean BP (95 % confidence interval, 95 % CI) of 4.18 mm Hg (1.03–7.33), 4.44 mm Hg (1.65 to 7.22) and 4.35 mm Hg (1.70 to 7.01) compared to normal trajectory, respectively. Trajectory 4 was significantly associated with SBP, DBP and MAP with mean BP of 6.08 mm Hg (2.93 to 9.23), 4.28 mm Hg (1.50 to 7.07) and 4.88 mm Hg (2.23 to 7.54) compared to normal weight trajectory, accordingly. Girls in Trajectories 3 and 4 had a 1.95 fold (1.01 to 3.77) and a 2.18 fold (1.31 to 4.20) increased risk of elevated BP in late adolescence, separately.

**Table 4:** The association of BMI trajectories, birth weight and height and blood pressure at 18 years for girls

	Unadjusted	Adjusted
SBP (mm Hg)		
BMI Trajectory		
Normal Weight	Reference	Reference
Late Onset Overweight	1.77 (−0.38 to 3.93)^a^	2.07 (−0.08 to 4.21)
Early Onset Obese to Overweight	3.66 (0.50 to 6.82)*	4.18 (1.03 to 7.33)**
Early Onset Obese to Morbidity Obese	5.87 (2.71 to 9.03)**	6.08 (2.93 to 9.23)**
Birth Weight (kg)		−0.61 (−1.99 to 0.78)
Height (cm) at 18 years		0.22 (0.11 to 0.34)**
R^2^	0.02	0.04
DBP (mm Hg)		
BMI Trajectory		
Normal Weight	Reference	Reference
Late Onset Overweight	1.33 (−0.57 to 3.22)	1.40 (−0.50 to 3.30)
Early Onset Obese to Overweight	4.29 (1.52 to 7.07)**	4.44 (1.65 to 7.22)**
Early Onset Obese to Morbidity Obese	4.25 (1.48 to 7.03)**	4.28 (1.50 to 7.07)**
Birth Weight (kg)		−0.05 (−1.27 to 1.18)
Height (cm) at 18 years		0.06 (−0.04 to 0.16)
R^2^	0.02	0.02
MAP (mm Hg)		
BMI Trajectory		
Normal Weight	Reference	Reference
Late Onset Overweight	1.47 (−0.33 to 3.28)^a^	1.62 (−0.19 to 3.43)
Early Onset Obese to Overweight	4.08 (1.43 to 6.73)***	4.35 (1.70 to 7.01)***
Early Onset Obese to Morbidity Obese	4.79 (2.14 to 7.44)***	4.88 (2.23 to 7.54)***
Birth Weight (kg)		−0.23 (−1.40 to 0.93)
Height (cm) at 18 years		0.12 (0.02 to 0.21)*
R^2^	0.03	0.03
Elevated BP at 18 years (Normal BP-reference)	OR	OR
BMI Trajectory		
Normal Weight	Reference	Reference
Late Onset Overweight	1.13 (0.70 to 1.82)	1.14 (0.71 to 1.84)
Early Onset Obese to Overweight	1.93 (1.00 to 3.72)*	1.95 (1.01 to 3.77)*
Early Onset Obese to Morbidity Obese	2.15 (1.11 to 4.12)*	2.18 (1.31 to 4.20)*
Birth Weight (kg)		0.93 (0.67 to 1.27)
Height (cm) at 18 years		1.01 (0.98 to 1.03)
Pseudo R^2^	0.01	0.01

*SBP* systolic blood pressure, *DBP* diastolic blood pressure, *MAP* mean arterial pressure, *OR* odds ratio

Adjusted for birth weight and height at 18 years

*P < 0.05; **P < 0.01; ***P < 0.001

^a^Intercept (MAP), OR (elevated BP) and 95 % Confidence Interval are presented

**Table 5:** The association of BMI trajectories, birth weight and height and blood pressure at 18 years for boys

	Unadjusted	Adjusted
SBP (mm Hg)		
BMI Trajectory		
Normal Weight	Reference	Reference
Early Onset Overweight to Normal Weight	3.23 (−0.08 to 6.54)^a^	2.85 (−0.43 to 6.13)
Early Onset Overweight to Obese	13.46 (6.75 to 20.17)**	13.45 (6.82 to 20.07)**
Birth Weight (kg)		−1.35 (−2.85 to 0.14)
Height (cm) at 18 years		0.27 (0.15 to 0.38)**
R^2^	0.02	0.05
DBP (mm Hg)		
BMI Trajectory		
Normal Weight	Reference	Reference
Early Onset Overweight to Normal Weight	0.95 (−1.69 to 3.59)	0.844 (−1.80 to 3.49)
Early Onset Overweight to Obese	5.77 (0.41 to 11.13)	5.74 (0.39 to 11.10)*
Birth Weight (kg)		−0.53 (−1.7 to 0.68)
Height (cm) at 18 years		0.07 (−0.2 to 0.17)
R^2^	0.01	0.01
MAP (mm Hg)		
BMI Trajectory		
Normal Weight	Reference	Reference
Early Onset Overweight to Normal Weight	1.71 (−0.77 to 4.19)^a^	1.51 (−0.96 to 3.99)
Early Onset Overweight to Obese	8.33 (3.3 to 13.37)***	8.31 (3.30 to 13.32)***
Birth Weight (kg)		−0.80 (−1.93 to 0.33)
Height (cm) at 18 years		0.14 (0.05 to 0.22)**
R^2^	0.02	0.03
Elevated BP at 18 years (Normal BP-reference)	OR	OR
BMI Trajectory		
Normal Weight	Reference	Reference
Early Onset Overweight to Normal Weight	1.38 (0.74 to 2.55)	1.34(0.72 to 2.50)
Early Onset Overweight to Obese	2.38 (0.66 to 8.49)	2.39 (0.67 to 8.57)
Birth Weight (kg)		0.89 (0.66 to 1.18)
Height (cm) at 18 years		1.02 (1.00 to 1.05)*
Pseudo R^2^	0.003	0.01

*SBP* systolic blood pressure, *DBP* diastolic blood pressure, *MAP* mean arterial pressure; *OR* odds ratio

Adjusted for birth weight and height at 18 years

*P < 0.05; **P < 0.01; ***P < 0.001

^a^Intercept (MAP), OR (elevated BP) and 95 % Confidence Interval are presented

For boys, Trajectory 3 was significantly associated with SBP, DBP and MAP with mean elevated BP of 13.45 mm Hg (6.82 to 20.07), 5.74 mm Hg (0.30 to 11.10) and 8.31 mm Hg (3.30–13.32) compared to normal weight trajectory respectively. In boys we observed a weak association for Trajectory 3 with a 2.39 fold (0.67 to 8.57) increased risk of elevated BP in late adolescence. The main results did not vary adjusting by birth weight, height at 18 years or both in girls. In boys, only the association between Trajectory 3 and DBP varied after adjusting by both weight and height at 18 years. The association was not significant in the unadjusted model while significant in the adjusted model.

## Discussion

There has been a growing interest in studying the heterogeneity in adiposity (BMI) developmental patterns in different populations over past years. Taking advantage of data-driven methods such as LCGMM we explored the distinct developmental patterns of BMI across childhood to late adolescence and its association with late adolescent blood pressure in black South African children. Our results confirm that there is heterogeneity in BMI trajectories in the study sample and that trajectories vary between boys and girls. A four-class BMI trajectory model may best represent heterogeneity in BMI developmental patterns in girls (normal weight, late onset overweight, early onset obesity to overweight and early onset obesity to morbidly obese) and a three-class model among boys (normal weight, early onset overweight to normal and early onset overweight to obese trajectory). These results are consistent with those reported by Hejazi and colleagues in Canadian children aged 2–8 years but with different age group and ancestry population [50]. Similarly, a study by Haga et al. reported that BMI trajectories varied between boys and girls in Japanese children aged between 0 and 12 years [51]. Ventura et al. [46] reported four distinct BMI trajectories in girls only. Few studies have focused on gender differences in BMI trajectories. American, Canadian, Australian and Dutch studies in children reported either three or four-class distinct BMI trajectories in both girls and boys [29, 41, 45, 52–57]. Individuals in trajectories characterized by high BMI trends had an increased risk of developing hypertension in late adolescence compared to those in a normal weight trajectory in the current study.

Prevalence of elevated BP in late adolescence was relatively higher in boys (39.43 %) than in girls (30.38 %). This prevalence is consistent with a recent study in urban South African children that reported the prevalence of prehypertension or hypertension ranging between 17.6 and 40.8 % [12]. Another study in youth aged 15 to 24 years in rural Ghana reported levels of prehypertension or hypertension with a prevalence of 37.4 %. The prevalence of prehypertension or hypertension (48.8 vs. 26.6 %) was higher in males than in females [17]. It should be pointed out that the prevalence in the current study was also higher than that previously reported in some studies within and outside South Africa. A study done by Makgae and colleagues in rural South African children aged 6 to 13 years found that 1.0 to 5.8 % of boys and 3.1 to 11.4 % of girls were hypertensive [58]. Moselakgomo et al. assessed BMI and blood pressure among adolescent school children aged 10 to16 years in Limpopo province, South Africa and the results revealed that prevalence of hypertension was between 2.3 and 5.9 % [59]. The following blood pressure prevalence has been reported in other African countries. In Brazzaville, Congo 24.3 % in girls and 16.6 % prevalence of hypertension in boys school children aged 5 to 18 years [60]. In Egyptian adolescents aged 11–19 years revealed that the prevalence rates of prehypertension and hypertension were 5.7 and 4.0 %, respectively [16]. The mechanisms of the observed sex differences in elevated BP in our study sample are not clear but may be attributed to sexual dimorphism as observed in other BP control programs [61] and also due to cardiovascular fetal programming as reported before [61, 62].

Girls in the early onset obesity trajectories were likely to have elevated SBP, DBP and MAP in late adolescence compared to those in a healthy trajectory. They displayed a 118 % increased odds of developing hypertension in late adolescence compared to those in the normal weight trajectory. Boys in early onset overweight to obese trajectory were likely to have a higher SBP, DBP and MAP in late adolescence. For instance boys in this trajectory had a 13 mm Hg mean SBP higher than those in a normal weight trajectory.

One longitudinal study on life-course adiposity and blood pressure in late adolescents has been done in Australian children [63], three of the seven adiposity trajectories were associated with elevated blood pressure at 17 years old of age. We report that being in an early onset obese or overweight trajectory was associated with increased risk of elevated BP in both girls and boys which is in agreement with other studies, although comparative studies were performed in a different population and different age range [63]. Girls in the early onset obesity to overweight BMI trajectory had reduced risk of elevated BP in late adolescence compared to those who were in early onset obesity to morbidity obese trajectory despite the fact that both groups had early onset obesity. This study was not an intervention study but it is important to study the mechanisms behind the weight loss in this group of girls. This could be important in reducing hypertension risk later in life.

The current study has several strengths; firstly, the longitudinal study in black South African children is a representative sample of South African children hence more relevant in understanding the different BMI trajectories in black South African children. Using LCGMM in identifying trajectories is an ideal analytical method since it involves a number of criteria in selecting the best fitting model and it predicts individual class membership from the available data and its ability to deal with missing at random data. The current results therefore clearly show individual heterogeneity in BMI development in boys and girls from five years to late adolescence. Our analysis was stratified according to sex and performed in a South African black population, which gives us an in-depth understanding of the difference in BMI developmental patterns in black boys and girls in South Africa. These results suggest that targeted intervention might be developed for individuals in high-risk trajectories. Limitations of our current study include the fact that the sample used in this study is from Soweto, South Africa and generalizability to other parts of Africa should be treated with caution. Adiposity trajectories explain only 5 % variation in late adolescent blood pressure and we do not know yet what the other factors are, that would explain the remaining variance. We speculate that the role of genetics and epigenetics could be important, as previously reported [64]. The role of genetics and epigenetics on adiposity and growth patterns in African populations is understudied and it calls for further investigation. A recent study by Kagura and colleagues in this cohort reported no association between blood pressure at 18 and alcohol consumption or smoking or both at 18 years in this population [65] and thus we did not include these variables within the models. Furthermore, we examined walking to school as a proxy of physical activity but this variable was not able to different among participants given that most participants are walking extensively during the day to and from school.

Due to the limited sample size we might not be able to capture all trajectories; this also influenced the observation that the high-risk trajectory in boys comprised of very few individuals that might influence association analysis.

## Conclusions

In this study, we identified distinct sex-specific trajectories. The early onset obesity or overweight trajectories are associated with elevated blood pressure in late adolescence. These results signify the importance to consider patterns of BMI development, especially at early stage of development, so that prevention strategies may be implemented to target those individuals in high-risk developmental patterns.

Our study has shown that patterns of adiposity could be a preferred predictor of future SBP, DBP, MAP and elevated BP in late adolescents compared to cross-sectional BMI measures. Furthermore, identification of childhood obesity can help with early identification of those at risk of developing elevated BP and other chronic diseases in adulthood.

## Abbreviations

BMI, body mass index; DBP, diastolic blood pressure; LCGA: latent class growth analysis; LCGMM: latent class growth mixture modeling; MAP, mean arterial pressure; SBP, systolic blood pressure
